# Complete and Draft Genome Sequences of Nine Lactobacillus sakei Strains Selected from the Three Known Phylogenetic Lineages and Their Main Clonal Complexes

**DOI:** 10.1128/genomeA.00082-18

**Published:** 2018-04-19

**Authors:** Valentin Loux, Gwendoline Coeuret, Monique Zagorec, Marie-Christine Champomier Vergès, Stéphane Chaillou

**Affiliations:** a MICALIS institute, INRA, AgroParisTech, Université Paris-Saclay, Jouy-en-Josas, France; b MaIAGE, Mathématiques et Informatique Appliquées du Génome à l’Environnement, INRA, Université Paris-Saclay, Jouy-en-Josas, France

## Abstract

We present here the complete and draft genome sequences of nine Lactobacillus sakei strains, selected from the entire range of clonal complexes from the three known lineages of the species. The strains were chosen to provide a wide view of pangenomic and plasmidic diversity for this important foodborne species.

## GENOME ANNOUNCEMENT

Lactobacillus sakei is a highly prevalent species found in meat and seafood products. It is used as a model organism for studying food fermentation, biopreservation, and spoilage. These different outcomes might depend on the food matrix and storage conditions but can also be strain dependent, as the species is known to exhibit high intraspecies diversity (1, 2).

Therefore, sequencing of the L. sakei genome was carried out to obtain at least two complete genome sequences from strains belonging to each of the three lineages previously shown by multilocus sequence typing (2). The remaining strains were sequenced as drafts. Furthermore, two complete large plasmids were fully sequenced to increase our knowledge of the plasmidic repertoire of the species, including the 70-kb plasmid pJ23A1 of the parent strain, L. sakei 23K (3).

The whole-genome sequencing of these strains was carried out by Eurofins MWG Operon Laboratories (Ebersberg, Germany) using either a mix of Sanger and pyrosequencing GS-FLX+ for complete genomes or Illumina MiSeq 2 × 150-bp paired-end libraries for draft genomes. Reads were assembled *de novo* by Velvet software (4). All contigs were aligned against the relevant 23K strain complete genome using progressiveMauve (5), and annotation was performed with a combination of MicroScope (6) and AGMIAL (7) platforms.

### Accession number(s).

Sequence data have been deposited in DDBJ/ENA/GenBank under the accession numbers cited in Table 1.

**TABLE 1:** Overview of the complete and draft genome assemblies from nine L. sakei strains

Phylogeny	Status	Original strain name (synonym[s])	Collection name	Source, yr	Genetic element	No. of contigs	Size (bp)	Coverage (×)	No. of CDSs^c^	BioProject no.	GenBank accession no.
Lineage 1^a^											
CC1A	Complete	J64 (64, JOUY-64)	CNCM I-4394	Dry sausage, 1982	Chromosome	1	2,050,232	49	2,102	PRJEB22335	LT960781
					Plasmid 2 (pJ64A2)	1	46,347	65	55		LT960782
					Plasmid 3 (pJ64F)	1	1,526	127	2		LT960783
CC1A	Complete	MFPB16A1401	CIP 110933	Beef carpaccio, 2009	Chromosome	1	1,994,583	51	1,960	PRJEB22338	LT960788
					Plasmid pMFPB16A1401A1	1	47,094	63	53		LT960789
Lineage 2											
CC2A	Complete	23K^d^		Dry sausage, 1990	Plasmid pJ23A1	1	70,516	51	92	PRJEB22564	LT907984
CC2B	Complete	MFPB19A1501 (MFPB19)	CNCM I-4396	Beef carpaccio, 2009	Chromosome	1	2,045,293	65	1,977	PRJEB22337	LT960784
					Plasmid 2 (pMFPB19A1501A1)	1	57,338	67	67		LT960785
					Plasmid 3 (pMFPB19A1501B)	1	11,996	154	16		LT960786
					Plasmid 4 (pMFPB19A1501C)	1	11,156	252	9		LT960787
CC2B	Draft	J156 (156, JOUY-156)	CIP 110931	Dry sausage, 1990	Chromosome	53	1,799,177	58	1,817	PRJEB22332	OBHL01000001– OBHL01000053
					Plasmid pJ156A1	1	48,944	86	55		OBHL01000054
					Plasmid pJ156B	1	12,002	299	15		OBHL01000055
					Plasmid pJ156C	1	10,871	237	9		OBHL01000056
					Plasmid pJ156D	1	6,593	343	7		OBHL01000057
					Plasmid pJ156E	1	2,855	501	4		OBHL01000058
CC2C	Draft	J160x1 (160x1)	CIP 110932	Horse meat, 1981	Chromosome	32	1,842,916	61	1,857	PRJEB22333	OBHK01000001– OBHK01000032
					Plasmid pJ160A1	1	51,764	504	62		OBHK01000033
					Plasmid pJ160E	1	2,211	441	4		OBHK01000034
Lineage 3											
CC3A	Complete	FLEC01	CNCM I-4395	Human feces, 2008	Chromosome	1	1,902,372	46	1,919	PRJEB22336	LT960777
					Plasmid 2 (pFLEC01A2)	1	31,701	55	36		LT960778
					Plasmid 3 (pFLEC01B)	1	12,663	345	16		LT960779
					Plasmid 4 (pFLEC01C)	1	11,068	377	10		LT960780
CC3A	Draft	J112 (112, JOUY-112)	CIP 110930	Dry sausage, 1990	Chromosome	38	1,893,060	85	1,913	PRJEB22330	OBHN01000001– OBHN01000038
					Plasmid pJ112A1	1	52,100	85	62		OBHN01000039
					Plasmid pJ112B	1	11,994	129	14		OBHN01000040
					Plasmid pJ112C	1	10,871	452	9		OBHN01000041
					Plasmid pJ112D	1	6,593	588	7		OBHN01000042
CC3B	Complete	J54 (54, JOUY-54)	CNCM I-4393	Dry sausage, 1990	Chromosome	1	1,964,671	76	1,982	PRJEB22334	LT960790
					Plasmid 2 (pJ54A1)	1	33,463	86	36		LT960791
					Plasmid 3 (pJ54C)	1	13,205	241	15		LT960792
CC3B	Complete	T332 (332)^e^		Pork meat, 1987	Plasmid p332A2	1	72,950	43	89	PRJEB22565	LT907987
Admixed^b^	Draft	J18 (18, JOUY-18)	CIP 110934	Dry sausage, 1990	Chromosome	27	1,824,746	56	1,826	PRJEB22331	OBHJ01000001– OBHJ01000027

a According to Chaillou et al. (2).

b Multilocus sequence typing previously revealed that strain J18 is a mixture of the three lineages due to numerous recombination events (2).

c CDS, coding sequence.

d Plasmid pJ23A1 originated from the strain L. sakei 23, the chromosome of which has already been sequenced (3).

e Plasmid p332A2 was sequenced from strain 332, the chromosome of which has been sequenced.
